# Fast and Accurate Charge State Deconvolution of Protein Mass Spectra

**DOI:** 10.1021/acs.analchem.5c00288

**Published:** 2025-07-07

**Authors:** Kenneth R. Durbin, Matthew T. Robey, Joseph B. Greer, Ryan T. Fellers, Aaron O. Bailey

**Affiliations:** † Proteinaceous, Inc., Evanston, Illinois 60201, United States; ‡ AbCellera Biologics, Inc., Vancouver, British Columbia V5Y 1G6, Canada

## Abstract

Charge state deconvolution is essential for efficient and effective protein mass spectrometry analysis. High-quality mass profiling is necessary to determine which proteoforms are present in protein samples and their relative abundances. In the pursuit of a well-rounded deconvolution solution, we detail an iterative charge state deconvolution algorithm named kDecon that has been tuned to provide high accuracy in its mass results while also delivering superb sensitivity toward lower abundance proteoforms in complex spectra. Here, the performance of kDecon as a mass determination algorithm for both targeted antibody and high-throughput proteomics analysis was benchmarked against existing deconvolution solutions. While the different deconvolution routines all proved robust for detecting the highest abundance protein species, kDecon ultimately showcased best-in-class precision for lower abundance proteoform mass profiling. Furthermore, kDecon results had up to 7-fold fewer false positives and simultaneously exhibited at least 20-fold speed improvements over the other algorithms. Overall, these deconvolution advances will contribute to enabling both routine and thorough intact mass profiling studies for biotherapeutics as well as improving the proteome coverage of top-down proteomics experiments.

## Graphical abstract

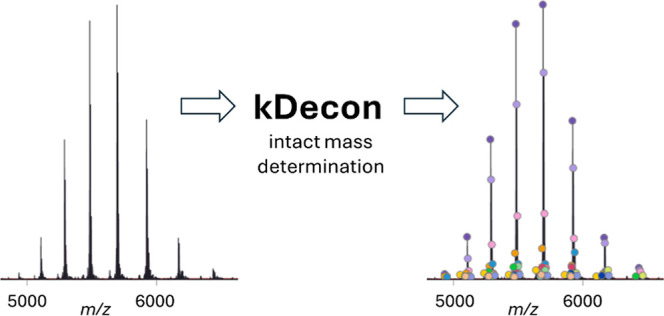

## Introduction

Mass spectrometry has been an important analytical technique for studying intact proteins and their associated proteoforms.^1,2^ Recent instrument improvements have enabled routine analysis of higher mass (>25 kDa) proteins and their associated proteoforms from complex mixtures.^3^ As the average mass of the human proteome is centered around 55 kDa and most biologic therapeutics exceed 100 kDa, these instrument advancements are key for improving the study of higher mass proteoforms.^4^

The common mass determination method for peptides and proteins is to use the *m*/*z* spacing between isotopologues within an isotopic distribution from an individual charge state in order to calculate the charge state and subsequently, the mass.^5^ Depending on the resolving power of the mass spectrometer, the individual charge states of the protein may not be isotopically resolved. For example, when masses exceed 25 kDa, achieving isotopic resolution of protein charge states becomes challenging, particularly at chromatographic time scales. Accordingly, another technique is needed to determine masses of isotopically unresolved proteins. Mass spectrometrists can instead focus on collecting spectra on large proteins with lower resolution and then perform charge state distribution deconvolution to obtain mass information.^6^ For instance, lower resolution data collected using shorter length transients in the Orbitrap have been shown to be more sensitive and exhibit higher signal-to-noise ratios than higher-resolution data collected with longer transients.^7^ While these data are generally not isotopically resolved, a well-designed deconvolution algorithm can still determine the masses of any multiply charged species present in the spectra with high accuracy that is typically in the low single digit parts per million errors when compared to the theoretical mass of the proteoform.

In theory, the simplest charge state deconvolution algorithm can apply a wide charge state range to every peak in a mass spectrum and end up with a list containing all of the correct masses; however, this would also likely produce an incredibly high number of false positives. As such, many charge state deconvolution algorithms have been developed over the years with different tactics for maintaining sensitivity of mass detection while striving to limit the number of false positives. Several algorithms have been developed to handle more industry-focused deconvolution applications such as antibody deconvolution. Among the most used are MaxEnt, Protein Metrics Intact Mass (PMI Intact), ReSpect, and UniDec.^8,9^ Other algorithms commonly used for deconvolution in top-down proteomics applications such as Xtract, THRASH, and TopFD do not have charge state deconvolution capabilities so cannot be used for lower resolution mass determination of larger proteins.^10,11^ The deconvolution tools listed for large intact mass determination have all been validated and used extensively. However, due to the extensive computations required to carry out the algorithms, they can be slow and also prone to false positives.^9,12^ Newer algorithms have been proposed to lessen analysis times through different computational approaches.^12^ We therefore were interested in seeing if a simpler, less computationally expensive approach could be undertaken to accelerate analysis times and limit incorrect assignments. Our starting place was our kDecon algorithm, a straightforward iterative charge state deconvolution tool, that was originally developed over 15 years ago to handle highly noisy ion trap data from early top-down proteomics experiments.^13^ These data had very high background noise levels that necessitated various algorithmic accommodations in order to differentiate signal from noise. As data from newer instruments is substantially different from those older ion trap data, particularly when looking at the low noise spectral data from lower resolution Orbitrap scans, we revisited the original kDecon algorithm to see how it could be modified to work with modern-day data.^7^ The original algorithm revealed several shortcomings when it came to the accuracy and sensitivity of results due to its simplistic nature and limited error checking. Following a complete rework of its internal algorithmic routines as well as substantial additions to limit false positives, we present a new and improved version of kDecon for protein mass deconvolution that offers heightened sensitivity and accuracy. Below, we detail the new algorithmic workings of kDecon as well as present different benchmark comparisons to other popular deconvolution tools on relevant workflows from both biopharma and academia. Overall, the new kDecon showcases sizable speed improvements together with increased sensitivity and selectivity over existing deconvolution tools.

## Methods

### Mass Spectrometry Data

All spectral data collected using Thermo Scientific mass spectrometers was obtained using Orbitrap systems run at a resolving power of 7500. NIST monoclonal antibody data was analyzed with denatured online buffer exchange (dOBE) coupled to an Agilent 6545-XT.^14^ NIST antibody was also analyzed by SampleStream (Integrated Protein Technologies) coupled to a Bruker maXis II instrument.^15^ Waters Intact mAb Mass Check was analyzed by LC–MS using a Sciex ZenoTOF 7600 mass spectrometer.^16^ Adalimumab data was collected in native mode via static nanoESI with a Thermo Scientific Q Exactive Plus.^17^ Lower mass LC–MS ribosomal data was collected using a Thermo Scientific Q Exactive HF-X instrument.^7^ High mass LC–MS proteomics data was collected on a Thermo Scientific Eclipse instrument using tPTCR.^18^ AAVs were collected by static nanoESI on a Thermo Scientific Q Exactive UHMR.^19^ Nucleosome data was obtained using a Thermo Scientific Q Exactive UHMR.^20^ Fab profiling data was obtained using LC–MS with a Thermo Scientific Fusion Lumos.^21^

### Spectral Charge State Deconvolution Algorithm

The charge state deconvolution algorithm kDecon was implemented using C# in .NET 8.0. The input is an array of *m*/*z* values and their corresponding intensity values. Noise, if present, is also specified in the same format. Typically for Thermo Scientific Orbitrap data, the sampled noise array from the spectral data in the raw file is used as the noise level. The kDecon algorithm is described in detail in the Results section.

### Deconvolution Analysis with kDecon

Our ProSight Native (v1.0.24275, Proteinaceous, Inc.) software was used for both targeted spectra and file-level kDecon sliding window analysis.^22^ The ‘Interactive Decon’ workflow was used to deconvolute individual spectra while the ‘Batch Decon’ mode was used for full file sliding window deconvolution analyses. Maximum charge state and maximum mass settings were set to accommodate the highest masses in each sample with additional clearance to test algorithmic robustness. For example, the settings for the deconvolution of intact antibodies used a minimum mass of 125 kDa and a maximum mass of 160 kDa to test for off-by-*n* assignments (i.e., off-by-*n* assignments of ≥3 on each side for an intact antibody centered at ∼3000 *m*/*z*). All proteins analyzed in this study had at least 5 charge states in their charge state distributions, so the parameter Low Number of Charge States was set to false for all analyses. For sliding window deconvolution, a merge tolerance of 10 Da was used with a retention time tolerance of 1 min. The minimum number of observations was set to 3. A window width of 0.2 min with an offset of 0.05 min was used for sliding window deconvolution.

### Deconvolution Analysis with ReSpect

BioPharma Finder (v5.2, Thermo Fisher Scientific) software was used in the ‘Intact Mass’ workflow for targeted spectra and file-level sliding window analysis by ReSpect. Multiple settings were attempted to optimize results, particularly in regard to the parameters for merging mass components. Ultimately, the ‘Legacy’ merge scheme with a tolerance of 50 ppm and a Max RT Gap of 1 min was selected due to the highest number of true positive results. The minimum number of observations (min. number of detected intervals) was set to 3. To match the kDecon sliding window settings in ProSight Native, a target avg. spectrum width of 0.2 min with a % Offset (legacy) of 25 was used. For ReSpect parameters, the minimum and maximum mass and charge state values were identical to kDecon. The Target Mass for the ReSpect Peak Model was set to a value near the expected masses of the analysis (e.g., 47,000 Da for the Fab analysis).

### Deconvolution Analysis with UniDec

The UniDec and UniChrom workflows of UniDec (v6.0.4) were used. Charge range and mass range were matched to kDecon settings during comparisons with the Sample Mass in UniDec set to every 1 Da. Smooth nearby points was set to ‘Some’ and Suppress Artifacts was set to ‘None’. The default settings were used for the additional deconvolution parameters. For peak selection, a peak detection range of 1 Da was used and the peak detection threshold was set between 0.001–0.01 and explicitly defined in the Results section.

### Deconvolution Analysis with PMI Intact

The Intact workflow in Byosphere (Protein Metrics, v5.6.66) was used with nearly all default settings, which were found to be effective for analyzing intact antibody samples. Deconvolution parameter settings that were modified from the default settings included a mass range of 143–163 kDa, a charge state range of 5–200, *m*/*z* range of 1000–4000, and a maximum number of reported species set to 100.

### Deconvolution Analysis with FLASHDeconv

Trastuzumab data was analyzed with the FLASHDeconv algorithm using the FLASHDeconv Wizard (OpenMS 3.1.0). The default settings were used except for the following modifications: min_isotope_cosine was set to 0, min_mass was set to 125,000, max_mass was set to 160,000, and min_charge was lowered to 50.

### Proteomics Data Analysis

Top-down proteomics data was analyzed using a developmental version of ProSightPD (Proteinaceous, Inc.) in Proteome Discoverer 3.2 (Thermo Fisher Scientific). The kDecon cRAWler was paired with an annotated proteoform search with 100 Da precursor mass tolerance. Data was searched against the entire Swiss-Prot UniProt database. A 1% FDR filter was applied to search results.

## Results

### Charge State Deconvolution with kDecon

Our charge state deconvolution algorithm, kDecon, is based on a straightforward iterative concept (Figure 1A). kDecon uses the top *n* abundant peaks as starting points for mass determination (Figure 1B). For each peak in the top *n*, the algorithm cycles through the possible charge states to find other peaks that correspond to correct *m*/*z* spacings for theoretical charge states of the candidate mass. Without any other considerations, this simplistic approach works well for mass spectral analysis in denaturing mode of simple mixtures. The spectra of single intact proteins are relatively easy to deconvolute (Figure S1) with clearly distinguishable charge state distributions such that even manual interpretation and mass determination is simple. However, as more mass species are present in a spectrum, the charge state distributions begin to overlap, creating a significantly more difficult informatics challenge. Furthermore, as the number of peaks above noise in the spectrum increases, the chances of finding multiple peaks with *m*/*z* spacing corresponding to a false positive mass increase greatly. The original kDecon was capable of deconvoluting simple spectra but struggled with complex spectra containing multiple proteoforms. Furthermore, the original algorithm needed to have charge state and mass ranges tailored by the user to values that were close to what was in the spectrum. To compensate for the inherent complexity of spectra with multiple proteoform species and limit the amount of user input required, several filtering and error correction steps were added into the kDecon algorithm to increase accuracy while limiting the number of false positive masses (Figure 1).

**Figure 1:**
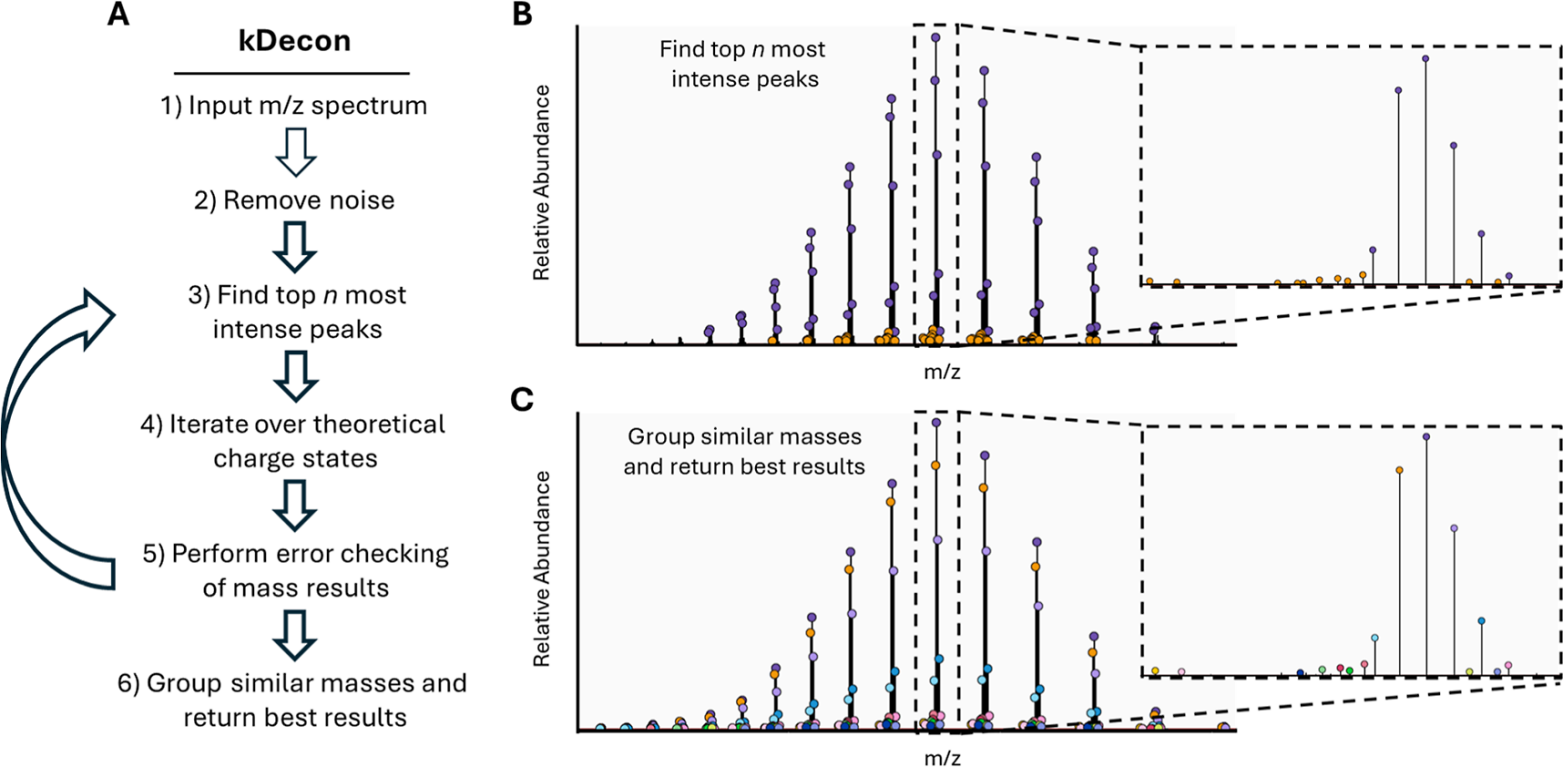
Overview of the kDecon spectral deconvolution algorithm for determining protein masses from charge state distributions. (A) The kDecon algorithm processes an input *m*/*z* spectrum, removes noise below the signal-to-noise-threshold, and then identifies the top *n* most intense peaks. Each of the top *n* peaks undergoes an iterative charge state assignment using all of the allowable charge states. During this process, other peaks within tolerance are grouped to form charge state distributions. Distributions with at least the minimum required number of charge states are then evaluated by an error-checking function to find harmonic masses and filter out poor fitting distributions. Peaks from mass distributions passing the error check are then subtracted and the algorithm performs another loop to look for additional masses using an updated top *n* peak list. Once complete, all masses are grouped within a defined mass grouping tolerance and the best mass results, ranked by iteration number and total intensity, are returned. (B) A representative spectrum from an antibody is shown with the top *n* peaks from two iterations highlighted in purple (first iteration) and yellow (second iteration). Here, *n* was set to 50. (C) The final determined mass results after performing a complete round of kDecon deconvolution are indicated with different color circles.

The steps of kDecon are as follows: First, a generic, user-defined signal-to-noise cutoff is applied to any input spectrum to separate signal peaks from noise. Any *m*/*z* values falling under the cutoff are removed from the spectrum that will be deconvoluted. The remaining *m*/*z* values are then formed into peaks and sent to the iterative charge matching function. kDecon sorts the peaks by intensity and takes the top *n* peaks as starting points for mass determination. Every top peak is assessed using the entire allowable charge state range. With each potential theoretical mass, kDecon attempts to match the corresponding *m*/*z* spacing to the other peaks in the spectrum on both the low and the high *m*/*z* side of the current starting charge state. Once a gap in the charge state distribution occurs, the algorithm moves on to the next possible theoretical mass from a different starting charge state. Once all top *n* peaks have been assessed over the full charge state range, filter and error correction steps are undertaken. kDecon initially checks the shape of the charge state distribution to ensure that no ‘jagged’ distribution is present; charge state distributions often get a jagged appearance when a harmonic mass has been matched (i.e., the found mass is an integer value multiple of the true mass or vice versa). While the jagged peak checking will catch many of the harmonic mass instances when neighboring charge states vary in a repeating way, there are instances where multiple large abundance differences are seen consecutively. Therefore, in addition to checks for harmonic assignments, the intensity variance between charge states is also assessed to prevent large differences in abundance between neighboring charge states as well as over stretches of charge states. The allowable variance in kDecon approximates a Gaussian distribution which has been previously observed to be a good approximation for charge state distributions.^23^ Additionally, a wide abundance deviation of up to 40% between individual charge states is included to account for signal variation among charge states. However, over the entire charge state distribution, the range cannot deviate significantly from the calculated theoretical value. If the mass fails at either one of the previous checks, a harmonic peak checking algorithm will attempt to find a harmonic mass that better fits the experimental data. If a harmonic mass is determined to be the valid result, then that harmonic mass will instead be returned as the mass result.

After the first deconvolution iteration, masses with similar molecular weights are grouped. All peaks belonging to the charge states of the grouped masses are then removed from the spectrum. The main goal of the subtraction routine is to reduce the number of peaks in the spectrum that could lead to false positives in subsequent deconvolution rounds. By only removing peaks belonging to confident results from the first iteration, the chance of removing peaks that were incorrectly assigned is substantially reduced. One or more additional iterations of the entire charge state matching process are then performed to look for more masses using a new list of top *n* peaks as starting points for mass determination. After all iterations are complete, the masses are grouped within a mass tolerance, ordered by iteration and total intensity, and then the top results are returned up to the maximum number of allowable results (Figure 1C).

### Robust Mass Determination from Complex Spectra Across Different Applications

Protein mass spectrometry can be used for many different applications to assess what proteins and proteoforms are present in a sample. The technique is applicable for proteins covering a wide range of molecular weights. Algorithmic versatility is therefore needed to handle the various forms of protein data. Additionally, simplifying user input is needed to make the deconvolution accessible by a user base with widely different experience levels. Before performing evaluations against other algorithms, we wanted to see if kDecon was capable of effectively analyzing data from different applications with minimal input needed. We therefore analyzed spectral data from several different protein samples, which tested the versatility of kDecon. Furthermore, we only changed the maximum mass setting for each sample to be relatively close to the top protein mass in the spectra, which was a fairly minimal amount of input from the user. First, we analyzed spectra with carbonic anhydrase (29 kDa) and adalimumab (148 kDa) that each had multiple proteoforms present of a single protein and were collected in denaturing mode and native mode, respectively (Figure 2A,B). The native mode shows the wider spacing between charge states due to lower charged species as well as fewer charge states in the charge state distributions, all of which is characteristic of proteins analyzed under native conditions. Conversely, the carbonic anhydrase analyzed in denaturing mode has many more charge states condensed into a much smaller spectral window, despite being ∼20% of the size of adalimumab. Manual interpretation confirmed that the algorithm was able to correctly find all the major species above a signal-to-noise of 3 that were present in both spectra without any false positives. Next, complex mixtures of proteins were analyzed for two different mass ranges with one range between 10 kDa and 30 kDa and the other range between 30 kDa and 80 kDa (Figure 2C,D). Manual interpretation was used to confirm the correct assignment of 10 proteoforms in Figure 2C and 9 proteoforms in Figure 2D, also with no false positives. To confirm that kDecon could also handle higher mass species, we also analyzed spectra containing multiple nucleosome species that were centered around 199 kDa as well as AAV spectra that were around 3.57 mDa (Figure S2). The main species were all found with kDecon and manually validated. Lastly, we wanted to ensure that kDecon could accurately assess data from non-Thermo instruments. We therefore analyzed antibody data obtained from Agilent, Bruker, and Sciex mass spectrometers (Figure S3). Despite differences in charge distribution, baseline levels, and peak shapes, kDecon was able to deconvolute the spectra with high accuracy and sensitivity. In particular for the Bruker data (Figure S3B), the spectra was complex, with multiple forms present including single light chain, intact antibody with glycosylation, intact antibody without glycosylation, and antibody without one light chain bound. The kDecon algorithm was able to successfully pick out all of these forms from the spectrum.

**Figure 2:**
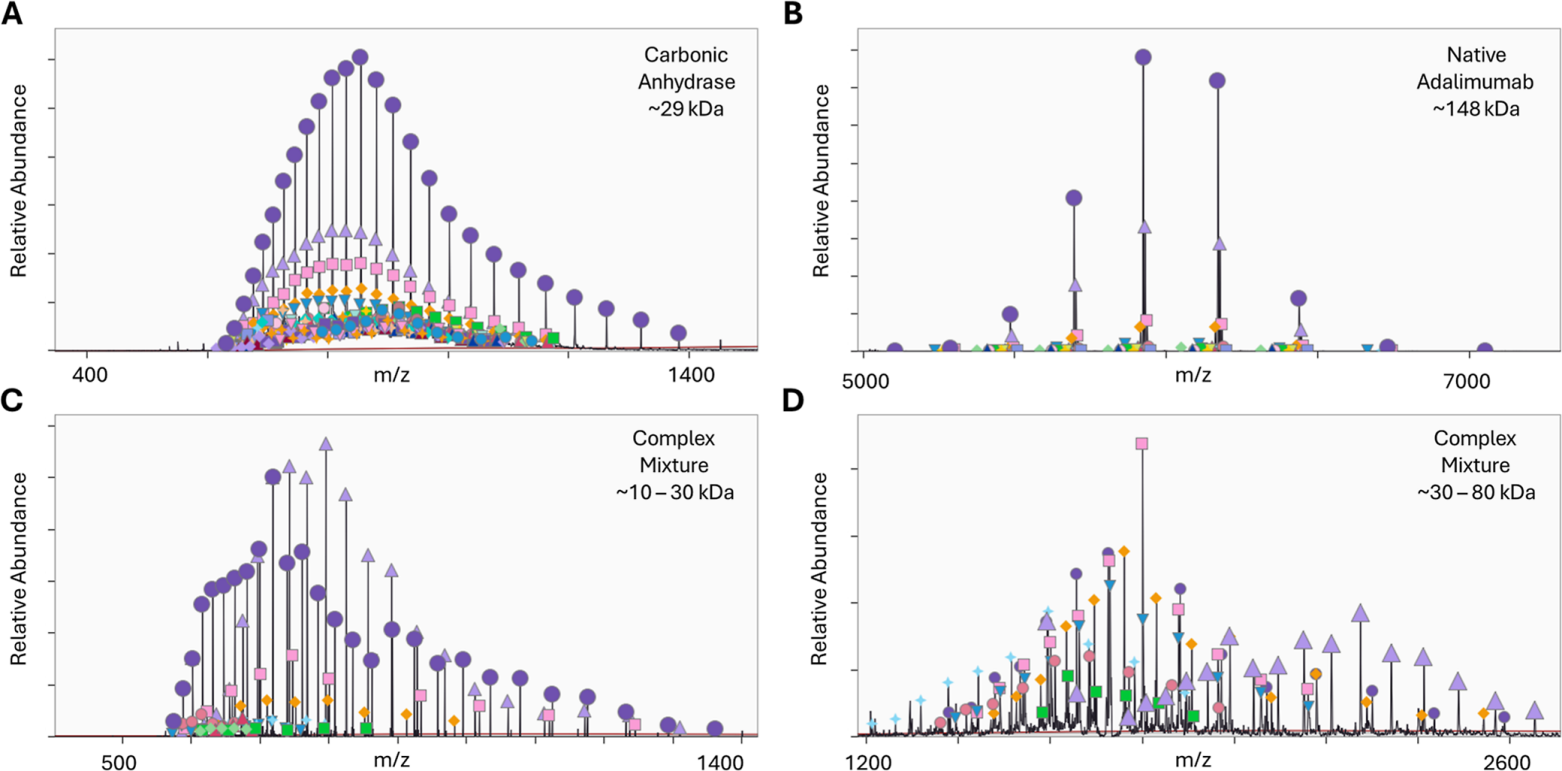
Mass determination using kDecon to deconvolute protein charge state distributions. Spectra from different protein samples were used to assess the versatility of kDecon including (A) oxidized carbonic anhydrase under denaturing conditions collected by direct infusion, (B) adalimumab glycoforms under native conditions, (C) a complex spectral mixture of proteins between 10 kDa and 30 kDa from an averaged window of LC–MS proteomics data, and (D) a complex spectral mixture of proteins between 30 kDa and 80 kDa from an averaged window of LC–MS proteomics data.

### Extracting Protein Modifications from Therapeutic Antibodies

An emphasis of kDecon’s routine is on deciphering proteoforms related to the most abundant proteins in a spectrum that we refer to as proteoform family members. The algorithm prioritizes forms that are similar in their charge state distributions to the major protein species over other possibilities. This structuring of the algorithm helps to both detect real modified forms that may be present at a lower abundance while also limiting potential false positives. In particular, false positives that are composed of distributions that have been shifted up or down by one or more charge states of true masses can be problematic for deconvolution routines in heavily modified spectra, as the charge states of the false positive masses move in a stair-step fashion across the proteoforms, landing on a different modification of the protein at each charge state. Herein, we refer to this type of false positive as an ‘off-by-*n*’ charge assignment result.

To benchmark the performance of kDecon, we analyzed a spectrum of intact trastuzumab where the signal was spread over many proteoform family members. We compared the output of kDecon to that of two widely used deconvolution platforms in PMI Intact and UniDec. Because PMI Intact does not have an implementation of overlapping sliding window deconvolution, we elected to analyze a single spectrum from the tail end of the chromatographic elution profile. This spectrum offered a balance of easier-to-deconvolute, higher abundance species as well as many lower signal-to-noise species; such a combination offered a valuable stress test of both sensitivity and selectivity for the three algorithms.

First, we compared the kDecon output to the PMI Intact output. The kDecon analysis yielded 98 distinct masses, all between 147 kDa and 149.5 kDa. PMI Intact produced 96 masses between a range between 145 kDa and 162 kDa. Comparing PMI Intact results to kDecon results, the 16 most abundant mass results were shared. Out of the remaining masses, 13 masses were matched, which equated to a total of ∼30% shared results between the two algorithms. Only 5 of the 67 remaining unmatched masses from PMI Intact were within the expected range of trastuzumab species in the sample. None of these 5 masses were able to be confidently verified through manual analysis. All of the other 62 masses from PMI corresponded to off-by-*n* distributions. The number of mass results produced by PMI Intact is largely dependent on a user-defined parameter that sets the maximum number of masses to be returned. As this parameter is increased, more masses are picked from the peaks of the output mass spectrum. We opted to set the number of masses value to a similar number as the masses output by kDecon in order to perform a quality comparison between the two sets of masses. This maximum number of masses parameter in PMI Intact could be lowered in order to generate fewer masses, which would generally increase the overall percentage of true masses but at the expense of potentially missing lower abundance species. We highlight that kDecon has a similar parameter but that setting it to a higher number typically does not result in an increase in false positives or off-by-*n* distributions. This attribute of kDecon reduces the amount of experience and time needed from a user to obtain high-quality deconvolution results.

To compare kDecon to UniDec, we performed an initial analysis with UniDec of the centroided version of the single spectrum with a conservative peak detection threshold (PDT) of 0.01. The analysis yielded 14 true masses that matched to the highest abundance species. Two false positive harmonic masses at half the expected mass were also returned. Changing from 0.01 PDT to 0.005 PDT increased the number of results from 16 to 25 masses. Manual analysis confirmed 18 of the masses to be antibody masses while 7 masses were false positives. After analyzing the spectrum with different settings for the PDT, we found that UniDec appeared to have a high reliance on the PDT parameter in terms of how many masses were generated as well as the accuracy of the mass results. Lowering PDT increased the number of results that were returned. However, these additional masses appear to be found at the expense of accuracy. For example, when changing the PDT from 0.005 PDT to 0.001 PDT, a significantly larger number of results were returned, with a ∼6× increase to yield 152 total mass results. Despite the increase in number of results, only 19 of these 152 masses fell within the range of the trastuzumab forms present in the spectrum while the rest spanned a wide range of results, from results under 10 kDa to many results with off-by*-n* distributions both above and below the expected trastuzumab masses.

The output masses from kDecon were all within the expected range for intact trastuzumab. However, from an accuracy standpoint with charge state deconvolution, only analyzing a single spectrum at a time has inherent difficulties. For example, several of the peaks in the data were assigned multiple masses by kDecon. Upon closer manual inspection, these masses had the same charge state distributions but with slight differences in the selected peaks at several of the charge states. The differences in the selected peaks were typically due to a split peak that caused minor mass differences (Figure S4). We made the design choice in kDecon to err on the side of reporting too many masses by including these instances, as a more complete sliding window deconvolution analysis can remove artifacts or instances where noisy peaks lead to single occurrences of additional mass assignments. Because sliding window deconvolution typically requires a minimum number of observations to include a mass, masses derived by chance from noise peaks would be eliminated after applying the minimum observations filter. For maximum confidence, a top-down mass spectrometry experiment could be carried out to determine how many of the peaks are real and the proteoform details of each. To aggregate all the results from the three algorithms into a single visual, we mapped the PMI and UniDec results to the most abundant charge state using matching kDecon masses to see the relative spectral peak coverages (Figure S5). All masses in this 49+ charge state range produced by PMI Intact and UniDec were also found in kDecon.

While the results from UniDec and PMI Intact were quite accurate for higher abundance species, they both had a number of false positive masses that stemmed from off-by-*n* charge state assignments. Prioritizing the finding of proteoform family members by the kDecon deconvolution routine allows for the algorithm to substantially reduce and even eliminate off-by-*n* assignments. While this type of prioritization can be accomplished in any charge state deconvolution algorithm by reducing the charge state range and mass range to that of the main protein species in the spectrum, such an approach requires both knowledge of the masses and user input. Additionally, if a diverse set of proteins are present, limiting the charge state and mass range will not work. There also stands the possibility that an unexpected truncation or modification to the protein could push the masses out of the small mass or charge state space being analyzed. Instead, kDecon can use wider parameter ranges and then automatically prioritize ranges that are best suited for the particular spectra. Conversely, both PMI Intact and UniDec appeared to be quite sensitive to the user-selected parameters that controlled the number of masses being generated from their mass spectra. Furthermore, the speed differences when moving from PMI Intact to UniDec to kDecon were significant. When recreating sliding window analysis using a similar number of multiple time slices and a maximum of 100 reported species for each slice, the file analysis took ∼20 min to process with PMI Intact run with Byosphere cloud, while UniDec runtime was ∼21 s and kDecon runtime was <1 s on a standard PC running a 13th generation Intel i7 CPU. We would like to note that the more standard way of processing the file in PMI Intact would be to either manually select the highest abundance peak or to use their peak picking routine. From this file, a single peak took ∼2 min to process in Byosphere. However, as we detail below, a sliding window routine can offer improved selectivity over single spectrum deconvolution.

### Deconvolution of Top-Down Proteomics Spectra

Accurate protein deconvolution also plays a key role in top-down proteomics applications. A central tenet of creating a reliable and efficient top-down proteoform search is having access to accurate precursor masses. These masses limit the scope of the database search for each mass target. With accurate mass assignments, one can use tight candidate proteoform window tolerances centered around input precursor masses. Smaller windows reduce the total number of proteoform candidates being considered at search time, which decreases both search times and false positives.

To test the utility of kDecon for providing precursor masses in a top-down proteomics context for larger proteins without isotopic resolution, we implemented kDecon as the mass detection algorithm in one of our ProSightPD ‘cRAWler’ nodes in the Proteome Discoverer platform. The cRAWler node is tasked with averaging scans, performing deconvolution of precursor and fragmentation scans, and associating precursor masses with their corresponding fragmentation data. For a direct comparison of high-resolution scans using an isotope determination algorithm against lower resolution scans with kDecon charge state deconvolution, we looked at a previously published ribosomal data set with a file containing high-resolution precursor scans and a file containing lower resolution precursor scans.^7^ The high-resolution file was cRAWled with Xtract, while the lower resolution precursor file was cRAWled with kDecon. Overall, the results were highly similar with 55 ribosomal protein accession groups found by the Xtract cRAWler and 55 ribosomal protein groups found with the kDecon cRAWler. All proteins were shared between the sets except for 30S ribosomal protein S1 being found in the lower resolution file and 50S ribosomal protein L31 type B being found in the higher resolution file. Notably, the S1 is a 61 kDa protein that is too large to be isotopically resolved under standard chromatography conditions, highlighting an advantage of collecting data in lower resolution mode on Orbitrap systems. Without charge state deconvolution, analyzing higher mass proteins by top-down proteomics becomes extremely difficult, and in effect artificially lowers the proteome coverage achievable by modern day mass spectrometers. One additional benefit of using lower resolution Orbitrap scans is also a lower duty cycle that leaves significantly more time for collection of fragmentation data.

To extend the identification comparisons to a slightly larger data set, we additionally analyzed the human ribosomal data set from the same publication that consisted of five runs analyzing the 40S and 60S ribosomal subunits. Compared to the published results, kDecon-led cRAWler results were able to identify 75 distinct ribosomal proteins. Both the high-resolution and ReSpect cRAWler results from the study led to 64 ribosomal proteins each with 57 proteins shared between the two. Comparatively, kDecon identified 70 of the 71 total proteins found by the combined high-resolution and ReSpect cRAWler results, along with an additional 4 proteins that were not previously identified.

The ribosomal data set provided a solid starting point for comparing how kDecon compares to existing charge state and isotopically resolved algorithms. However, because the protein identification results were so similar, we needed a more complex data set. We therefore followed this comparison by next looking at complex samples from both a proteomics application as well as a biopharma application.

### Finding Multiple Mass Species

Related to the challenge of extracting protein modifications within proteoform family members that was described above is the parsing of spectra that contains many different proteins within a narrow mass range. To test the performance of kDecon with these types of data, we analyzed spectra detailing the Fab profiles from human serum antibody repertoires.^21^ These spectra contained mass species between ∼47–49 kDa with fairly uniform charge state distributions. Here, we focused on two spectra with one spectrum containing higher abundance masses and the other spectrum containing lower abundance masses. We compared the deconvolution results from kDecon to the results from ReSpect and UniDec. For the higher abundance spectrum, the kDecon algorithm returned 26 masses in the expected mass range. All masses were manually verified. With a 0.01 PDT, UniDec had 12 masses matching within 10 Da to the kDecon mass output and 4 masses that were off-by-*n* masses. One other mass at 48,145 Da was within 16 Da of one of the lower abundance kDecon mass results but after manual analysis, did not appear to be a real result. ReSpect matched 8 masses from the kDecon output that corresponded to the more abundant masses in the spectrum. Additionally, ReSpect produced 23 masses that were off-by-*n* results on the high mass side and 20 masses that were off-by-*n* results on the low mass side. For the lower abundance spectrum, kDecon returned 16 masses. There was one mass at 49,089 Da that shared two charge states with lower mass species, making it difficult to confirm as a correct mass and not an off-by-*n* error. However, this mass had several other charge states that did not match anything else, increasing the confidence in the assignment. UniDec returned 21 masses when using a PDT of 0.01 with 13 masses matching to the kDecon output masses. Of the other 8 masses, 2 were not able to be validated manually. There were also 4 masses that potentially corresponded to real masses but were difficult to confirm as well as 2 masses not originally found by kDecon but that could be detected by kDecon if the signal-to-noise ratio was lowered to 1.0 (Figure S6). ReSpect found 21 masses with 9 matching kDecon masses, 9 corresponding to off-by-*n* results, 1 that was incorrect, and 2 that were potentially real but not high confidence mass assignments.

To compare results with a larger data set, we next performed a sliding window deconvolution comparison between ReSpect and kDecon. The use of a sliding window approach during spectral deconvolution has proven to be immensely powerful for analyzing protein mass spectra.^24^ Deconvoluting multiple spectral windows across an elution peak and requiring a minimum number of observations serves to both provide enough points across the peak for quantitation while also greatly reducing false positive mass assignments. For the comparisons, the ReSpect result set was obtained by using the well-established sliding window deconvolution implementation in BioPharma Finder (BPF). The kDecon result set was obtained using the sliding window algorithm in ProSight Native. All mass observations meeting the minimum observations criteria were rolled up into mass components.

Overall, using the sliding window algorithm in ProSight Native, kDecon detected 488 mass components with a merge tolerance of 10 Da. Using the sliding window algorithm in BPF, ReSpect detected 3207 mass components with a merge tolerance of 50 ppm. We assessed different merge tolerances in BPF in an attempt to match parameters. Larger tolerance values in ReSpect yielded far fewer overall masses but at the expense of returning high-quality results. We therefore settled on the lower merge tolerance of 50 ppm for ReSpect to provide the highest quality comparison. 463 mass components from kDecon matched within 10 Da and 1 min RT to ReSpect sliding window results. Of the 488 total kDecon mass components, 20 masses were >49 kDa and 27 masses were <46 kDa for a total of ∼10% belonging to masses outside of the expected Fab mass range. There were 7 masses above 0.5% relative abundance that were unique to kDecon and all were manually verified to be real masses. Of the 3207 total ReSpect mass components, 73% of the results belonged to masses higher or lower than expected Fab masses with 1079 results >49 kDa and 1269 results <46 kDa. There were 13 masses above 0.5% relative abundance that were unique to ReSpect. Following manual inspection, 9 were deemed to be off-by-*n* masses, 3 were deemed to be correct, and 1 was inconclusive. After additional checking, kDecon was able to find 2 of the correct masses in at least one spectrum but not enough overall observations to count as a valid sliding window result. Overall, the dichotomy in the ReSpect data between the high number of accurate mass determinations together with a high number of false positive hits experimentally highlights the importance of charge state deconvolution algorithms striking a balance between sensitivity and accuracy. From these data, it appears kDecon is able to greatly reduce the number of false positives while still maintaining the ability to uncover a high percentage of real proteoforms in the sample (Figure 3).

**Figure 3:**
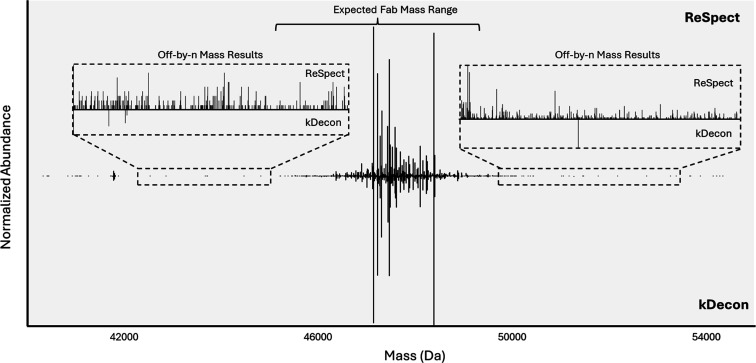
Sliding window deconvolution of human Fab repertoire. The mass spectrum from the mass components of a sliding window deconvolution run across an entire file are shown in a mirror plot. ReSpect masses from BioPharma Finder are shown on top and kDecon masses from ProSight Native are shown on the bottom. All masses were normalized to the most abundant mass from each respective algorithm’s output. The expected range of Fab masses is between ∼46 kDa and ∼49 kDa. Off-by-*n* charge state assignments result in slightly lower or higher mass assignments. Regions with likely off-by-*n* masses are shown with zoomed-in spectral windows.

### Sliding Window Deconvolution Reproducibility Across a Data Set

We next processed a data set of 10 replicate injections of intact trastuzumab that were analyzed by an Orbitrap using lower resolution (i.e., unresolved isotopic distributions) to see the reproducibility of sliding window deconvolution across files. For the comparisons, ReSpect in BPF, kDecon in ProSight Native, and a newer sliding window implementation in UniDec were all used. There were 7 proteoform family member masses that corresponded to common glycoforms (Figure 4A). Our main goal with the comparison was to see the reproducibility of the algorithms for finding these 7 trastuzumab proteoforms in the data as well as their overall reported abundances with associated standard deviation.

**Figure 4:**
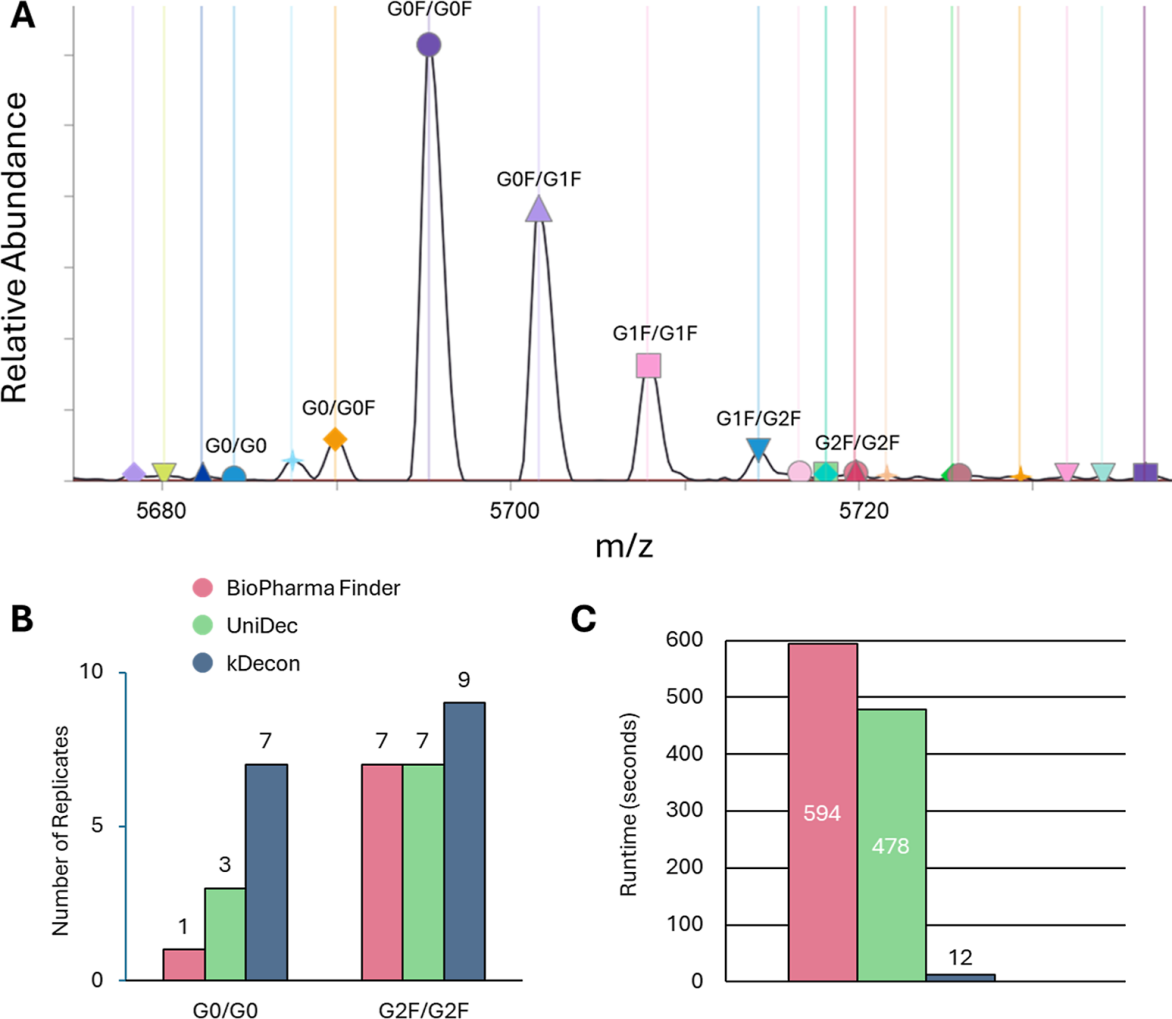
Deconvolution of intact trastuzumab variants. (A) kDecon mass deconvolution results are mapped onto the highest charge state of the distribution with the seven main glycoforms annotated. (B) The number of replicates where the low abundance glycoforms G0/G0 and G2F/G2F were observed and (C) the analysis runtimes of sliding window deconvolution were compared between ReSpect in BioPharma Finder, UniDec in UniChrom, and kDecon in ProSight Native.

For the top five major glycans, the three software tools were in agreement regarding the relative order of glycoforms when ranked by abundance. The other two forms corresponded to the G0/G0 glycoform and the G2F/G2F glycoform present at relative abundances of ∼1% and 2%, respectively, compared to the highest abundance form in the sample. For the minor form G0/G0, we observed a substantial increase in the number of replicates found by ProSight Native compared to BioPharma Finder and UniDec (Figures 4B and S7), with the minor form found in 7 replicates with kDecon versus 1 and 3 for BPF and UniDec, respectively. Using our targeted approach previously described,^25^ we were able to find the G0/G0 proteoform in one additional sample replicate. For the minor G2F/G2F form, ProSight Native found the form in 9 out of 10 replicates, which was two replicates more than that found by BPF and UniDec (Figure 4B). Regarding runtimes for sliding window of these 10 files, we observed a very sizable time difference between the software tools. BPF and UniDec completed the analyses in 594 and 478 s, respectively, while ProSight Native was able to complete the analyses in 12 s (Figure 4C). These times corresponded to an average of ∼1 s per data file for ProSight Native and represents an analysis rate 40× and 50× faster than UniDec and BPF.

### Comparison to Speed-Centric Deconvolution Routine

Seeing that the run times during this study have shown kDecon to be significantly faster than other deconvolution options, we sought out another algorithm that has been tailored for speed, FLASHDeconv. In our final comparison of this study, we processed the same 10 replicate injection data set as above with both kDecon and FLASHDeconv. Instead of sliding window deconvolution, only single scans were processed. The runtime of FLASHDeconv was ∼80 s versus the 4 s for kDecon. We did want to note that there may be a CPU-threading issue in the version of FLASHDeconv that we used as increasing the thread count did not lower the run time. The kDecon alternatively took advantage of multithreading the analysis in the ProSight Native application. Regarding data result quality, the FLASHDeconv output had a very wide range of masses, despite the limited range of masses present in the sample. For a single file, there were over 2826 results with 6 results in the mass range for trastuzumab variants.

## Conclusion

Mass deconvolution is a critical tool in many applications being deployed across academia and biopharma, including biomarker quantitation, quality control, biologics screening, and pharmacokinetics studies. In this study, we benchmarked the kDecon deconvolution algorithm against other popular deconvolution tools such as PMI Intact, ReSpect, and UniDec. Overall, we showed improvements to accuracy and sensitivity of protein mass deconvolution while also being able to significantly reduce analysis times. The speed improvements were primarily driven by the algorithm design, which consists of an iterative approach that cycles through possible charge states for each of the top *n* peaks in a spectrum. In contrast to the maximum entropy-based deconvolution routines, an iterative approach is much less computationally intensive. However, an iterative approach by itself is not sufficient as an algorithm for deconvoluting complex protein spectra. Therefore, our improvements in result quality were achieved through the use of extensive error checking routines that checked mass results for realistic charge state distributions and removed false positives. Accurate and sensitive deconvolution of intact protein mass spectra can greatly increase the value of information being obtained from protein mass spectrometry experiments. More accuracy in mass determination increases confidence in results, limits the chances of missing key proteoforms, and reduces the amount of manual validation required. While kDecon performed well for the applications shown here, the approach may not be suitable for all applications. Very complex molecules with poor peak resolution (e.g., polydisperse particles) may be better represented as wide peaks in a mass spectrum output than being reported as discrete masses.

As experimental techniques continue to be developed for high-throughput applications, such as protein–ligand screening by mass spectrometry, there becomes real value in having ultrafast algorithms for data processing. For instance, a recent study from AbbVie showed capabilities for analyzing an entire 384-well sample plate in ∼20 s.^26^ Another workflow from Merck using acoustic ejection mass spectrometry was able to screen for protein–ligand binding on >10,000 compounds in 17 h.^27^ At the deconvolution speeds of certain algorithms, data analysis can quickly become the bottleneck in a workflow. With the low millisecond run time of kDecon, deconvolution can easily keep pace with data collection, even at extremely rapid acquisition rates seen with newer TOF systems. Furthermore, with the analysis speed increases demonstrated here, kDecon could be integrated into future real-time processing routines to guide advanced instrument data acquisition for more informed and productive top-down proteomics data collection.^28^ Accurate deconvolution also helps improve the automated targeting of different protein species during acquisition by decreasing the number of times the same protein is targeted for fragmentation, ultimately leading to more time for selecting previously unfragmented proteoforms or characterizing proteoforms that require more sequence coverage. Altogether, improved deconvolution stands to increase the thoroughness and reproducibility of intact mass profiling experiments as well as bolstering future top-down proteomics data acquisition and search.

## Supplementary Material





## Data Availability

The data are available at the MassIVE repository under the accession MSV000097766. Data used here that were previously published are listed in the Supporting Information. Software availability: The kDecon algorithm is accessible through the software tools ProSightPD and ProSight Native. Free 60 day demo licenses for both tools are available. More information is available at proteinaceous.net.

## Notes

Additional figures with deconvoluted spectra and quantitation results and tables with data availability and deconvolution comparisons (PDF)
(XLSX)
