# Supplementary material for: Fast and Accurate Charge State Deconvolution of Protein Mass Spectra
Source: Anal Chem. 2025 Jul 7;97(28):14964–73. doi: 10.1021/acs.analchem.5c00288 (PMC12291047; doi:10.1021/acs.analchem.5c00288)
Supplement: Supplementary file 1 [file ac5c00288_si_001.pdf]

Fast and Accurate Charge State Deconvolution of Protein Mass Spectra

Kenneth R. Durbin[1*], Matthew T. Robey[1], Joseph B. Greer[1], Ryan T. Fellers[1], Aaron O. Bailey[2]

[1]Proteinaceous, Inc., Evanston, IL 60201, United States

[2]AbCellera Biologics, Inc., Vancouver, British Columbia V5Y 1G6, Canada

*Corresponding Author – Kenneth Durbin, kdurbin@proteinaceous.net

Tables of Contents for Supplemental Information

Supplemental Figure 1 – Simple spectrum with a single protein charge state distribution……………………………….2

Supplemental Figure 2 – High mass species deconvolution…………………………………………………………3

Supplemental Figure 3 – Deconvolution by kDecon of different instrument data……………………………………..4

Supplemental Figure 4 – Slight peak variations in kDecon results…………………………………………………….5

Supplemental Figure 5 – Differences in charge state annotations between deconvolution algorithms……….............6

Supplemental Figure 6 – Masses detected from UniDec that were not found by kDecon……………………………..7

Supplemental Figure 7 – Glycoform observations……………………………………………………………………..8

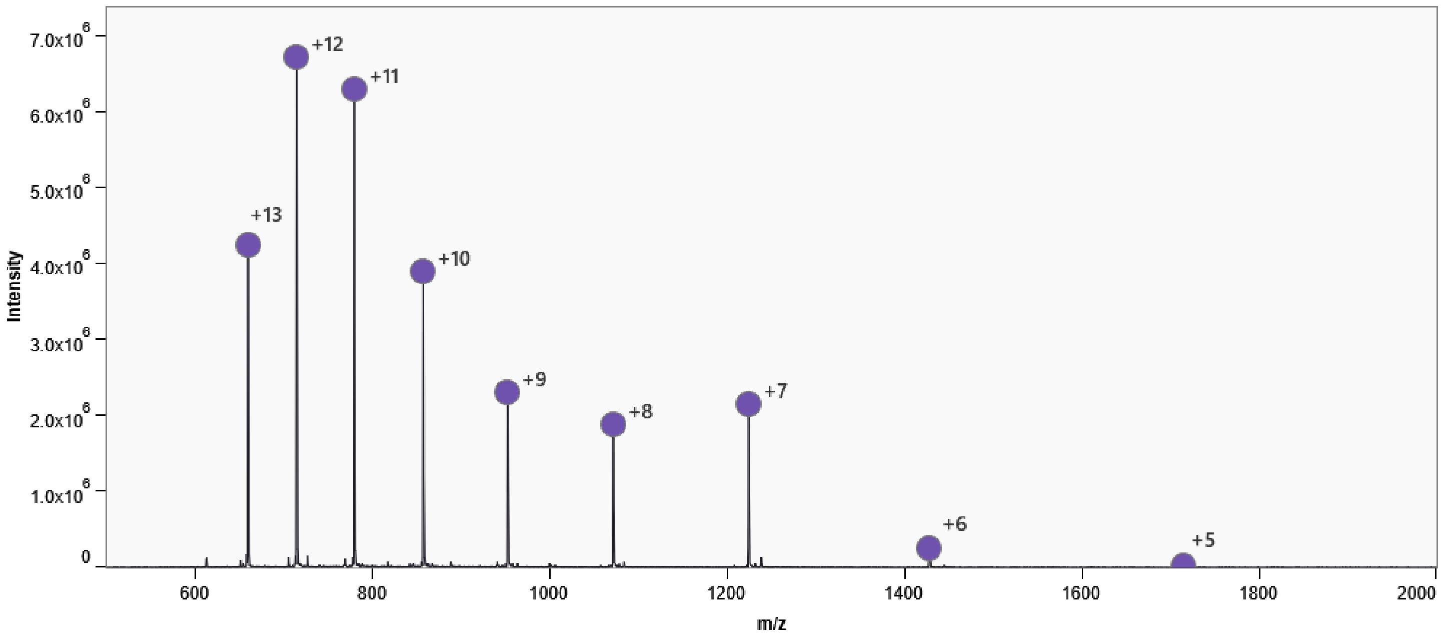

Supplemental Figure 1 – Simple spectrum with a single protein charge state distribution. The charge state distribution of ubiquitin using lower resolution Orbitrap scans is shown with the individual charge states annotated by purple circles.

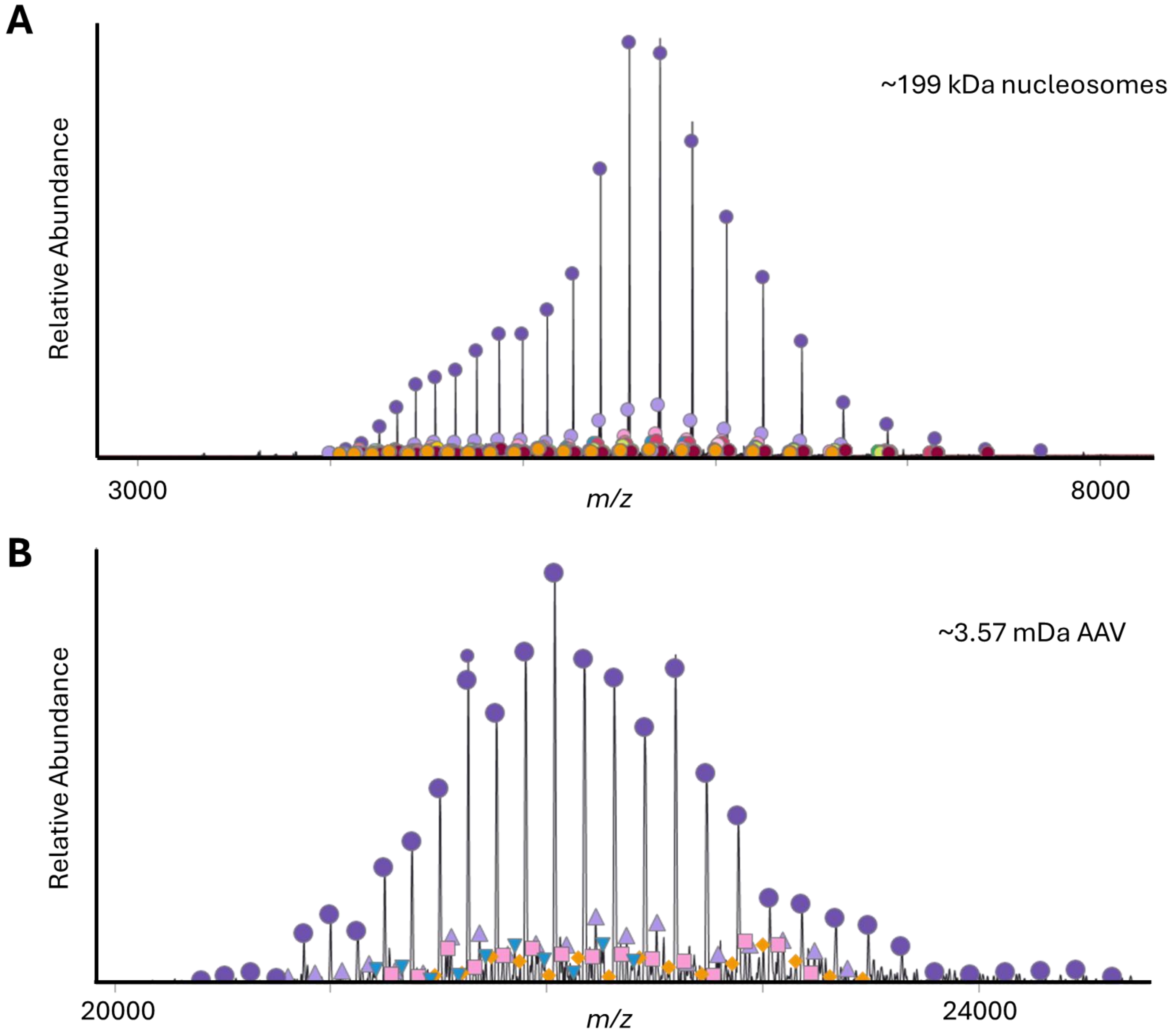

Supplemental Figure 2 – High mass species deconvolution. (A) Nucleosome data was deconvoluted by kDecon to reveal multiple nucleosome species in the range of 199 kDa. (B) AAV data was analyzed by kDecon with the deconvoluted species calculated to be 3.57 mDa.

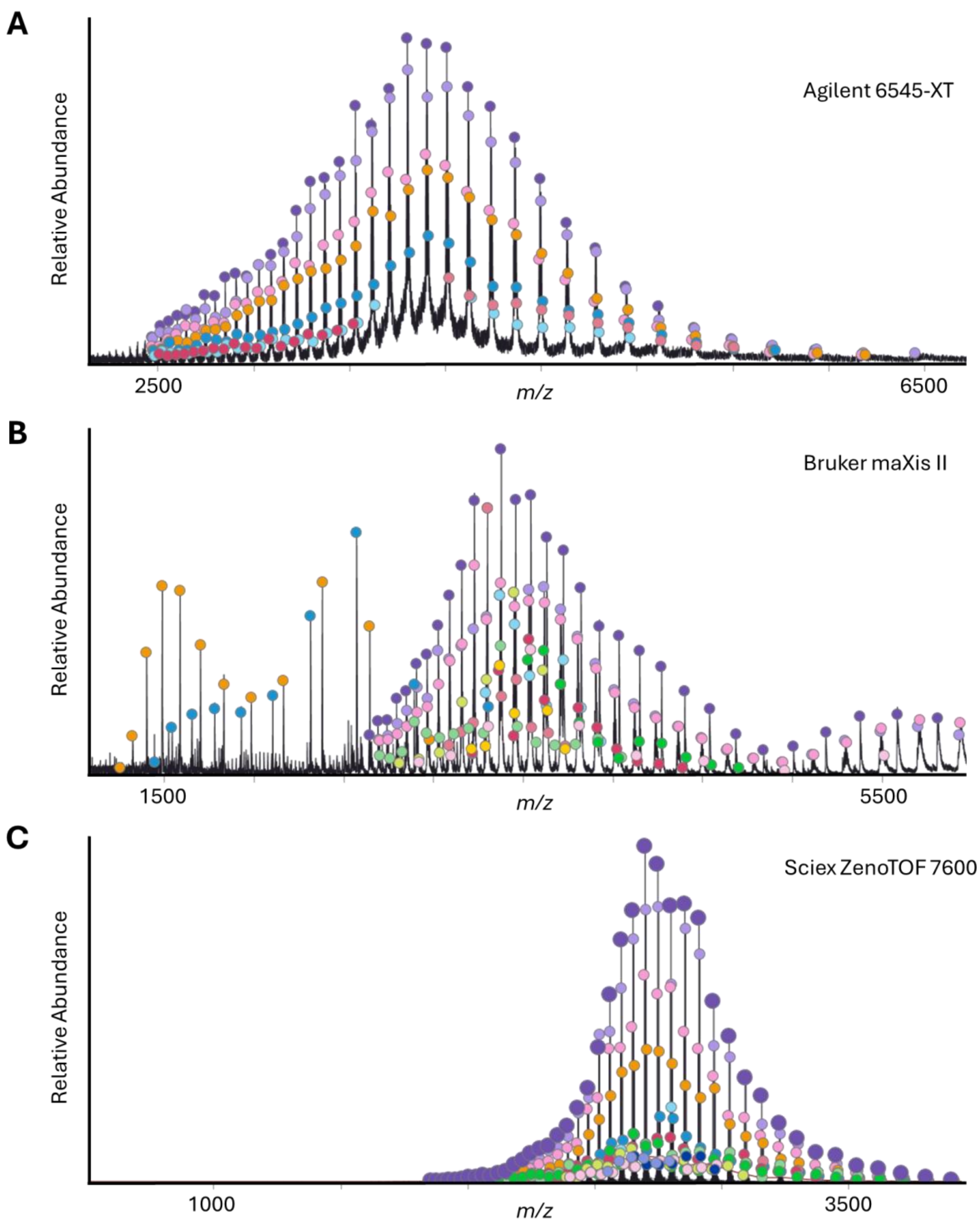

Supplemental Figure 3 – Deconvolution by kDecon of different instrument data. (A) NISTmAb was analyzed using online buffer exchanged and an Agilent 6545-XT mass spectrometer. (B) NISTmAb was analyzed using SampleStream and a Bruker maXis II mass spectrometer. (C) Waters Intact mAb Check was analyzed using LC-MS with a Sciex ZenoTOF 7600 mass spectrometer.

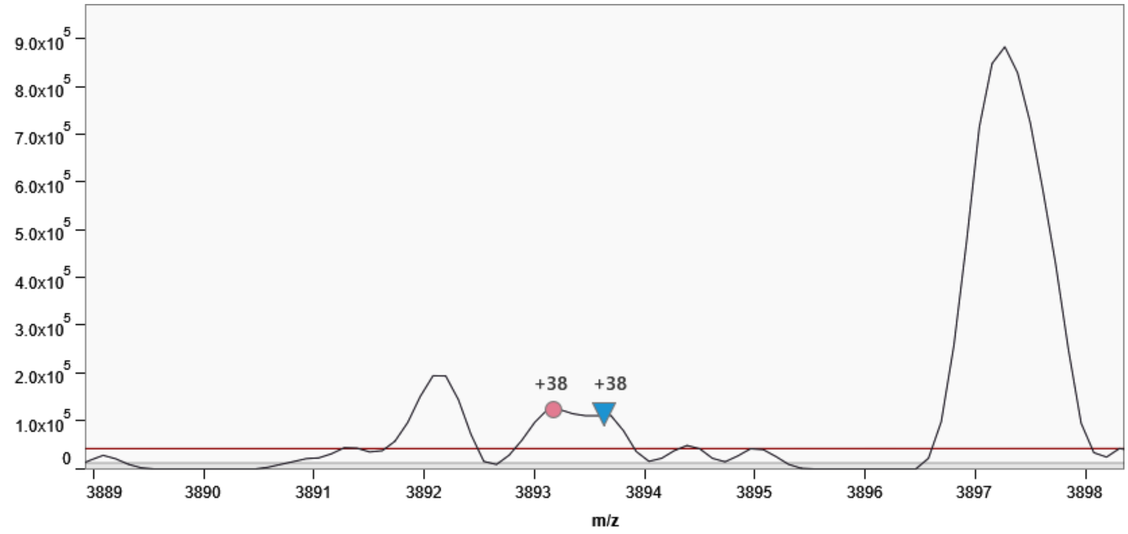

Supplemental Figure 4 – Slight peak variations in kDecon results. Occasionally, similar but slightly different mass assignments were returned from kDecon with overlapping identical charge states (i.e., the same charge state assignment on the same peak). Upon closer inspection, the mass differences were driven by minor variations in the makeup of the overall charge state distribution. Here, small differences in selected peaks due to the split peak for the 38+ charge state produced a mass deviation that resulted in two distinct masses that otherwise shared all other charge state peaks.

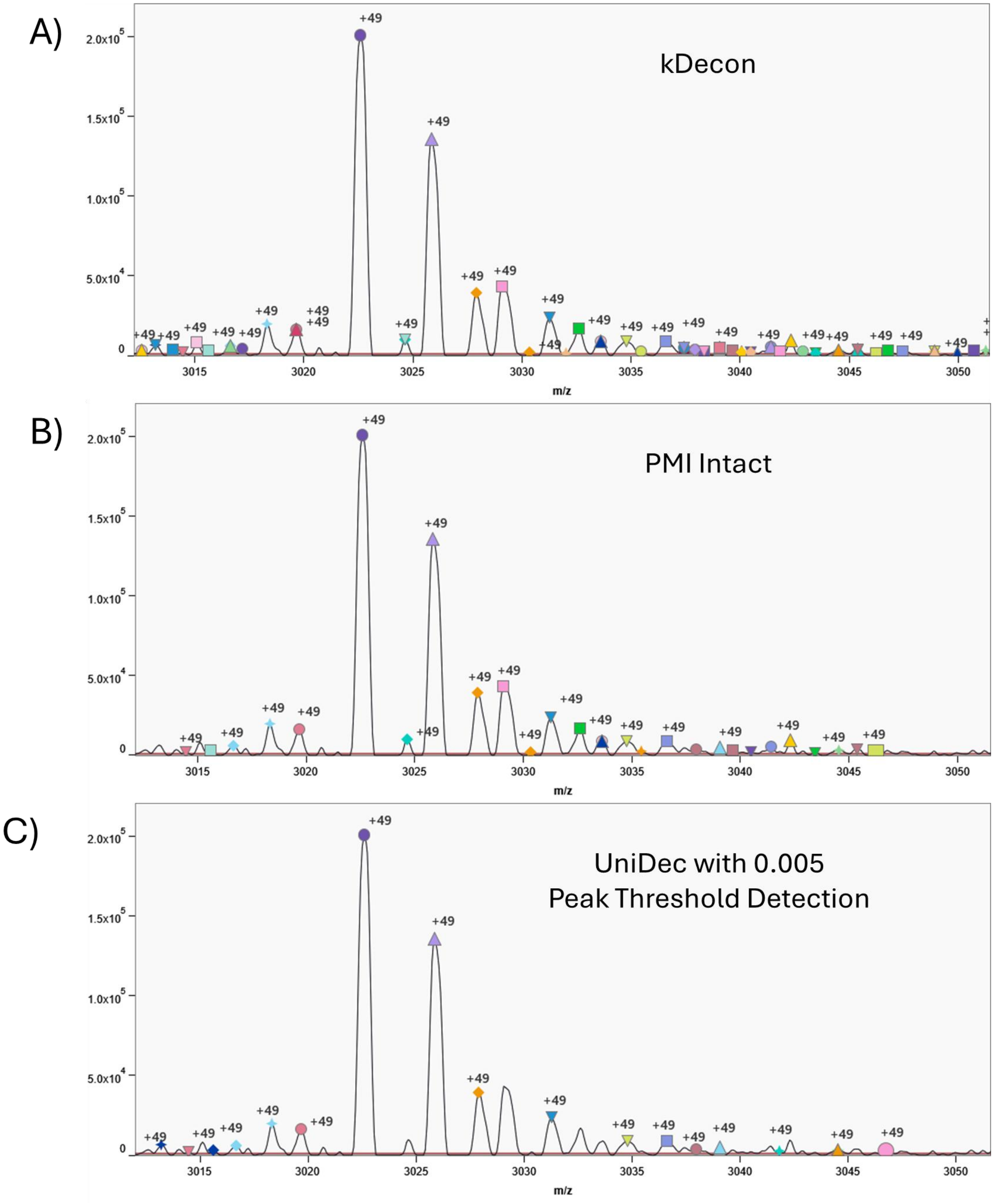

Supplemental Figure 5 – Differences in charge state annotations between deconvolution algorithms. The results from mass assessment of trastuzumab proteoforms by (A) kDecon, (B) PMI Intact, and (C) UniDec are shown for the 49+ charge state. The masses for PMI Intact and UniDec were mapped by selecting the closest kDecon output masses so that the colors and formats were the same for straightforward comparisons.

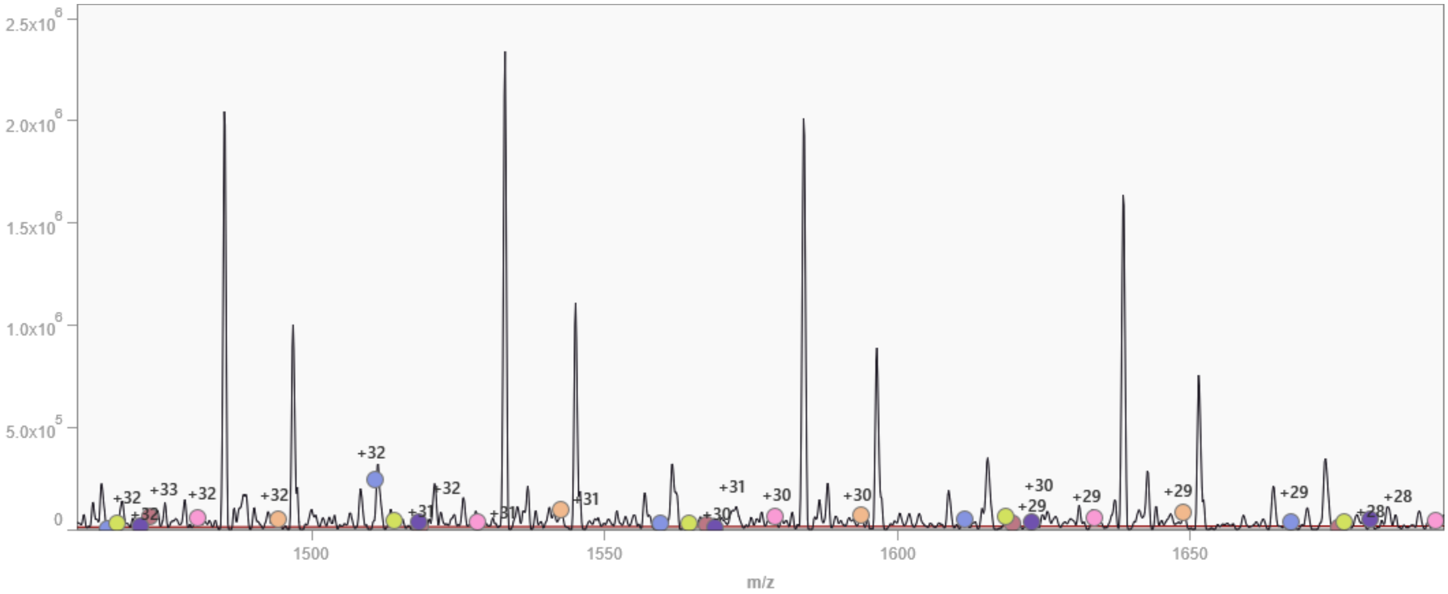

Supplemental Figure 6 – Masses detected from UniDec in Fab data that were not found by kDecon. There were 4 masses in this spectrum that were found by UniDec that were not able to be fully manually validated due to their low abundance and high variation in charge state signals. There were 2 other masses that were found by kDecon when the signal-to-noise threshold was lowered to 1.0 (pink and beige masses).

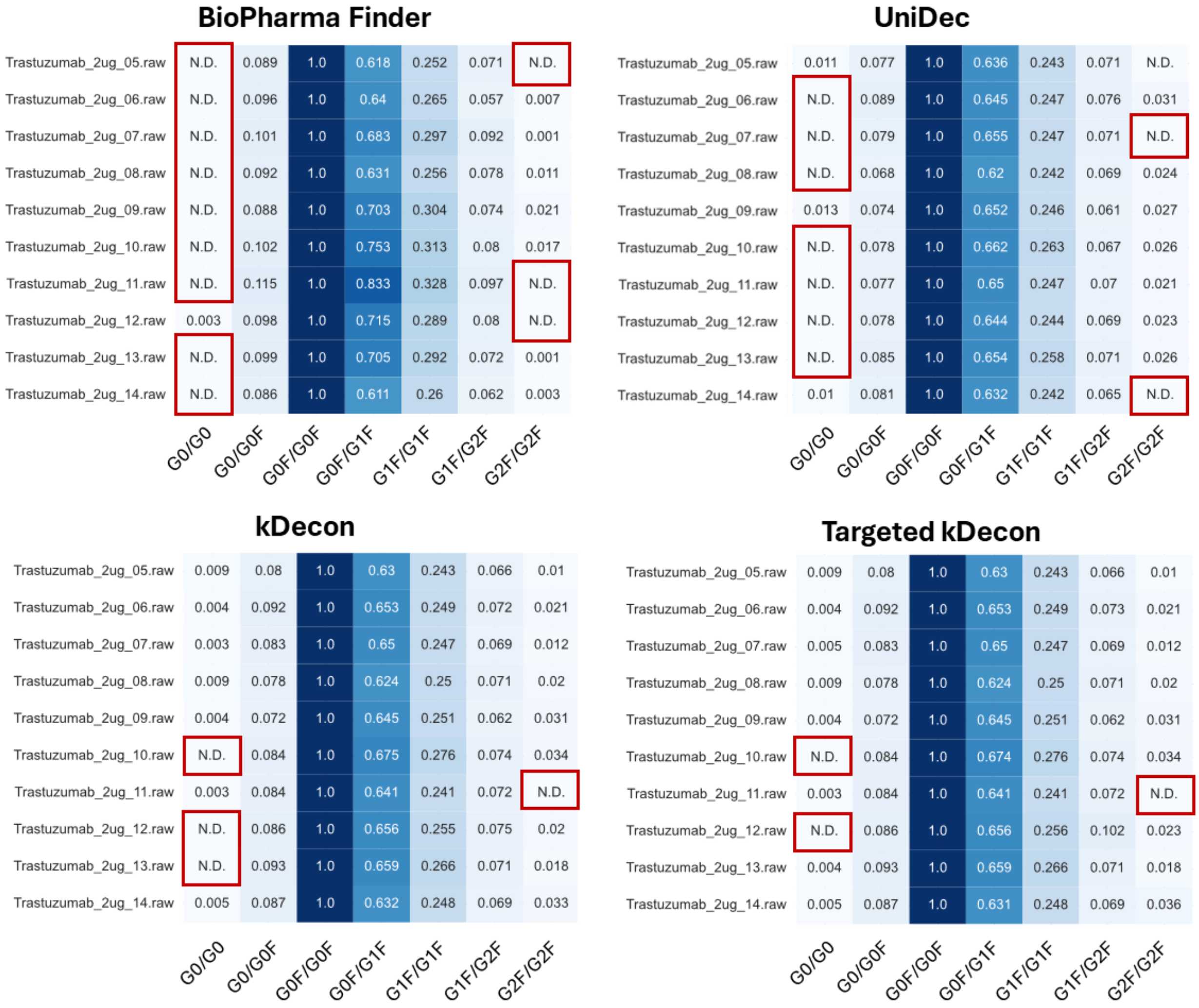

Supplemental Figure 7 – Glycoform observations. The observations of trastuzumab glycoforms across technical replicates for different sliding window deconvolution implementations are shown. Each column corresponds to a trastuzumab glycoform, and each row corresponds to a replicate injection. Cells outlined in red correspond to injections in which the corresponding glycoform was not detected by that algorithm.
